# Novel TCF4:TCF12 heterodimer inhibits glioblastoma growth

**DOI:** 10.1002/1878-0261.13496

**Published:** 2023-08-07

**Authors:** Svetlana A. Mikheeva, Cory C. Funk, Philip J. Horner, Robert C. Rostomily, Andrei M. Mikheev

**Affiliations:** ^1^ Department of Neurosurgery, Center for Neuroregeneration Houston Methodist Research Institute Texas USA; ^2^ Institute for Systems Biology Seattle Washington USA; ^3^ Department of Neurosurgery University of Washington Seattle Washington USA; ^4^ Institute for Stem Cell and Regenerative Medicine University of Washington Seattle Washington USA

**Keywords:** E‐box, glioblastoma, heterodimer, periostin, TWIST1

## Abstract

TWIST1 (TW) is a pro‐oncogenic basic helix–loop–helix (bHLH) transcription factor and promotes the hallmark features of malignancy (e.g., cell invasion, cancer cell stemness, and treatment resistance), which contribute to poor prognoses of glioblastoma (GBM). We previously reported that specific TW dimerization motifs regulate unique cellular phenotypes in GBM. For example, the TW:E12 heterodimer increases periostin (*POSTN*) expression and promotes cell invasion. TW dimer‐specific transcriptional regulation requires binding to the regulatory E‐box consensus sequences, but alternative bHLH dimers that balance TW dimer activity in regulating pro‐oncogenic TW target genes are unknown. We leveraged the ENCODE DNase I hypersensitivity data to identify E‐box sites and tethered TW:E12 and TW:TW proteins to validate dimer binding to E‐boxes *in vitro*. Subsequently, TW knockdown revealed a novel TCF4:TCF12 bHLH dimer occupying the same TW E‐box site that, when expressed as a tethered TCF4:TCF12 dimer, markedly repressed *POSTN* expression and extended animal survival. These observations support TCF4:TCF12 as a novel dimer with tumor‐suppressor activity in GBM that functions in part through displacement of and/or competitive inhibition of pro‐oncogenic TW dimers at E‐box sites.

## Graphical abstract

TWIST1 (TW) is a pro‐oncogenic basic helix–loop–helix transcription factor and promotes the hallmark features of malignancy. TW dimerization with E12 increases periostin (*POSTN*) expression through binding to the regulatory E‐box consensus sequences. We identified a novel TCF4:TCF12 bHLH dimer with tumor‐suppressor activity in glioblastoma that functions in part through displacement of and/or competitive inhibition of pro‐oncogenic TW dimers at E‐box sites.

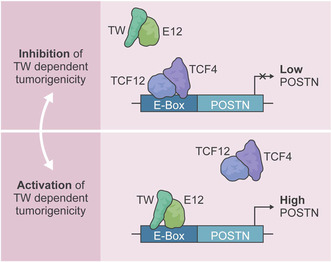

**Abbreviations**
bHLH: basic helix–loop–helix protein
BRDU: bromodeoxyuridine
DOX: doxycycline
EGF: epidermal growth factor
EMSA: electromobility shift assay
ENCODE: The Encyclopedia of DNA Elements
FDC: forced dimerization construct
FGF: fibroblast growth factor
GBM: glioblastoma
IP: immunoprecipitation
KD: knockdown
NE: nuclear extract
POSTN: periostin
PVDF: polyvinylidene membrane
TBE: Tris/borate/EDTA buffer
TCF12: transcription factor 12
TCF4: transcription factor 4
TF: transcription factor
TW: TWIST1

## 1 Introduction

TWIST1 (TW) is a basic helix–loop–helix (bHLH) domain, containing a transcription factor (TF) that promotes malignant phenotypes in a wide variety of tumors, including glioblastoma (GBM) [1, 2], head and neck [3], breast [4], bladder [5], gastric [6], hepatocellular [7], esophageal [8], nasopharyngeal [9] cancers, and chronic myelogenous leukemia [10] (for review [11]). TWIST1 expression levels directly correlate with a higher grade of cancer, local or distant metastasis, and/or unfavorable prognosis [12]. As a recognized master regulator of epithelial‐mesenchymal transition (EMT) in cancers, TW promotes canonical features of malignancy, including cell invasion, cancer cell stemness, and treatment resistance that contribute to poor prognoses [4, 11, 13, 14].

In prior studies, we identified TW as a critical regulator of GBM invasion, tumorigenicity, and glioma stem cell (GSC) phenotypes [2]. TWIST1 overexpression was sufficient to activate the invasiveness of nontumorigenic T98G cells *in vivo*, *ex vivo* in brain slices, and *in vitro* [2]. TWIST1 knockdown prolonged survival of mice xenografted with GSCs due to Caspase 3 activation *in vivo*, inhibition of cell invasion *in vitro*, and alteration of the extracellular matrix structure [1]. Furthermore, we demonstrated that inhibition of the direct TW transcriptional target gene, periostin (POSTN) [15] phenocopies the invasive and pro‐tumorigenic effects of TW loss of function on GBM malignancy [16]. Therefore, the mechanisms by which TW regulates POSTN have great significance for understanding how TW promotes GBM malignancy.

TWIST1 forms homodimers or heterodimers with other bHLH proteins that activate or repress target genes by binding to E‐box consensus recognition sites within regulatory regions [17]. We previously showed that different TW dimerization motifs have opposite effects on POSTN expression. Specifically, maximal expression of POSTN is dependent on TW:E12 heterodimers compared with TW:TW homodimers [18]. Of note, TW:E12 and TW:TW dimers differentially activate unique target gene profiles, indicating the general importance of TW dimerization motifs for defining transcriptional activity underlying malignant phenotypes.

In this study, we sought to define the transcriptional mechanisms of TW heterodimer‐specific activation of POSTN expression as a potential general model of TW‐dependent oncogenic activity. Using a repertoire of POSTN‐specific TW E‐box sites that were identified by analyzing The Encyclopedia of DNA Elements (ENCODE) data [19], we discovered a preferred E‐box site for TW:E12 binding. Using TW knockdown to validate the interaction, we unexpectedly identified a novel TCF4:TCF12 heterodimer that occupied the same site as TW:E12 in a state of TW deficiency. Functional characterization determined that TCF4:TCF12 not only occupied the POSTN TW E‐box site but also repressed POSTN expression and inhibited GBM malignancy *in vivo*. Together these data support the role of a novel bHLH heterodimer, TCF4:TCF12, that acts as putative tumor suppressor in part through competitive inhibition of TW:E12 E‐box binding.

## 2 Materials and methods

### 2.1 Bioinformatics

Transcription factor occupancy for over 1515 TFs was determined by using publicly available ENCODE DNase I hypersensitivity DNA‐seq data (https://www.encodeproject.org/) [19, 20]. Briefly, when a difference in read pile‐up was found to coincide with a known TF binding motif, a footprint was called. The motif library from MotifScan [21], which contains motifs for several E‐boxes, was utilized. Further details can be found in the aforementioned publications. We then queried for E‐box motifs ±5 kb from the transcriptional start site of POSTN.

### 2.2 Glioblastoma cells

Primary GBM4V cells derived from GBM patient were provided by Dr Wakimoto and cultured in serum‐free neurobasal media supplemented with EGF and FGF [22]. The STR profile for GBM4V cells does not match any profiles reported in Cellosaurus. U87MG (RRID: CVCL_0022) and T98G (RRID: CVCL_0556) cells were obtained from ATCC (Manassas, VA, USA) and cultured in DMEM/F12 media (Cytiva, Marlborough, MA, USA) supplemented with 10% Fetal Bovine Essence (VWR, Radnor, PA, USA). Cell identity was confirmed by short tandem repeat (STR) analysis of cells before experiment. Regular mycoplasma testing was performed using mycoplasma detection kit (Lonza Biologics, Houston, TX, USA).

### 2.3 Electromobility shift assay (EMSA)

EMSA was previously described [23]. Biotinylated oligonucleotides (Integrated DNA Technology, Inc., Coralville, IA, USA) and nuclear extracts (7 μg), isolated with hypertonic buffer per previous protocols [23], were mixed with binding buffer as recommended by Chemiluminescent EMSA kit (Active Motif, Carlsbad, CA, USA) to achieve final sodium chloride concentration of 150 μm. Control samples were mixed with bovine serum albumin. Binding reactions were supplemented with the antibodies (Table S1), incubated at room temperature for 30 min, and loaded on the gel. Where indicated, EMSA reactions were supplemented with a 30‐ or 100‐fold excess of wild‐type (WT) or mutant double‐stranded probe. After reaction separation on 5% Mini‐PROTEAN TBE precast polyacrylamide gels (Bio‐Rad, Irvine, CA, USA) and blotting, the nitrocellulose membranes (GE HealthCare, Chicago, IL, USA) were hybridized to streptavidin conjugated to horse‐radish peroxidase for 20 min, washed, and visualized using Chemidoc MP imager (Bio‐Rad).

### 2.4 Immunoprecipitation (IP)

Immunoprecipitation was previously described [18]. Western blotting analyses were performed using 4–15% gradient precast polyacrylamide gels (Bio‐Rad). Gels were blotted to the PVDF membrane (Bio‐Rad) and hybridized with indicated antibodies overnight [1, 2, 18, 24] (Table S1). fiji [25] software was used for digital gel image densitometry.

### 2.5 Lentiviral expression gene transfer

Lentiviral expression and knockdown DNA constructs (Table S2) were used to produce viruses and infect target cells, as we described previously [1, 18]. Forced dimerization construct (FDC) is composed of two monomer proteins fused in frame with flexible glycine‐rich linker.

### 2.6 Quantitative qRT‐PCR

Isolated tumor total RNA (1 μg) was reverse‐transcribed, and resulting cDNA was amplified using SYBR green PCR mix (Bio‐Rad) and predesigned primers spanning intron (Sigma‐Aldrich, St. Louis, MO, USA) as described [18].

### 2.7 Intracranial cell implantation

All animal studies were approved by Animal Care Committee of the Houston Methodist Research Institute and performed according to protocol IS00007124. Approximately equal numbers of 6–8‐week‐old male and female nude mice (NU/J, Jackson Laboratory, Bar Harbor, ME, USA) were used in the study. Animals were housed in standard cages using diet and water *ad libitum*. Preoperative care included approved anesthesia and local and systemic analgesia. Cells were injected stereotactically (U87MG‐80000 and GBM4V—150000 cells per animal) with aid of Cunningham™ Mouse adaptor (Stoelting, San Diego, CA, USA) and computer‐driven microinjector into the right brain hemisphere (1.8 mm lateral and 1.5 mm anterior relative to Bregma) [1, 16]. Where indicated, animals were switched to doxycycline supplemented chow, 625 mg·kg^−1^, (Envigo, Indianapolis, IN, USA) 5 days after cell implantation to activate doxycycline regulated TCF4:TCF12 FDC expression or control empty vector. Animals were sacrificed when developed clinical manifestation. To avoid animal suffering, the time of sacrifice was used as a surrogate for survival analysis. Tumor fragments were fixed in formalin for histological and immunohistochemical analyses. Where indicated, fresh tumor fragments were also snap‐frozen for subsequent RNA and protein extractions.

### 2.8 Tumor proliferation analysis

Tumor proliferation was analyzed by immunohistochemical staining of Ki67 antigen in paraffin‐embedded sections after antigen retrieval. The labeling index was determined as a percent of positive cells detected by nuclear counterstaining with hematoxylin. At least three independent and randomly selected tumor regions were analyzed per tumor.

### 2.9 Statistical analysis

For Kaplan–Meier studies, the statistical difference in median survival was determined by the log‐rank test (Mantel‐Cox) using GraphPad prizm version 9.5.0 software (Boston, MA, USA) at 95% confidence interval. For analysis of expression, two‐tailed *t*‐test was used to compare two groups.

## 3 Results

### 3.1 Validation of E‐box sequences identified in POSTN for TW binding

**Fig. 1:**
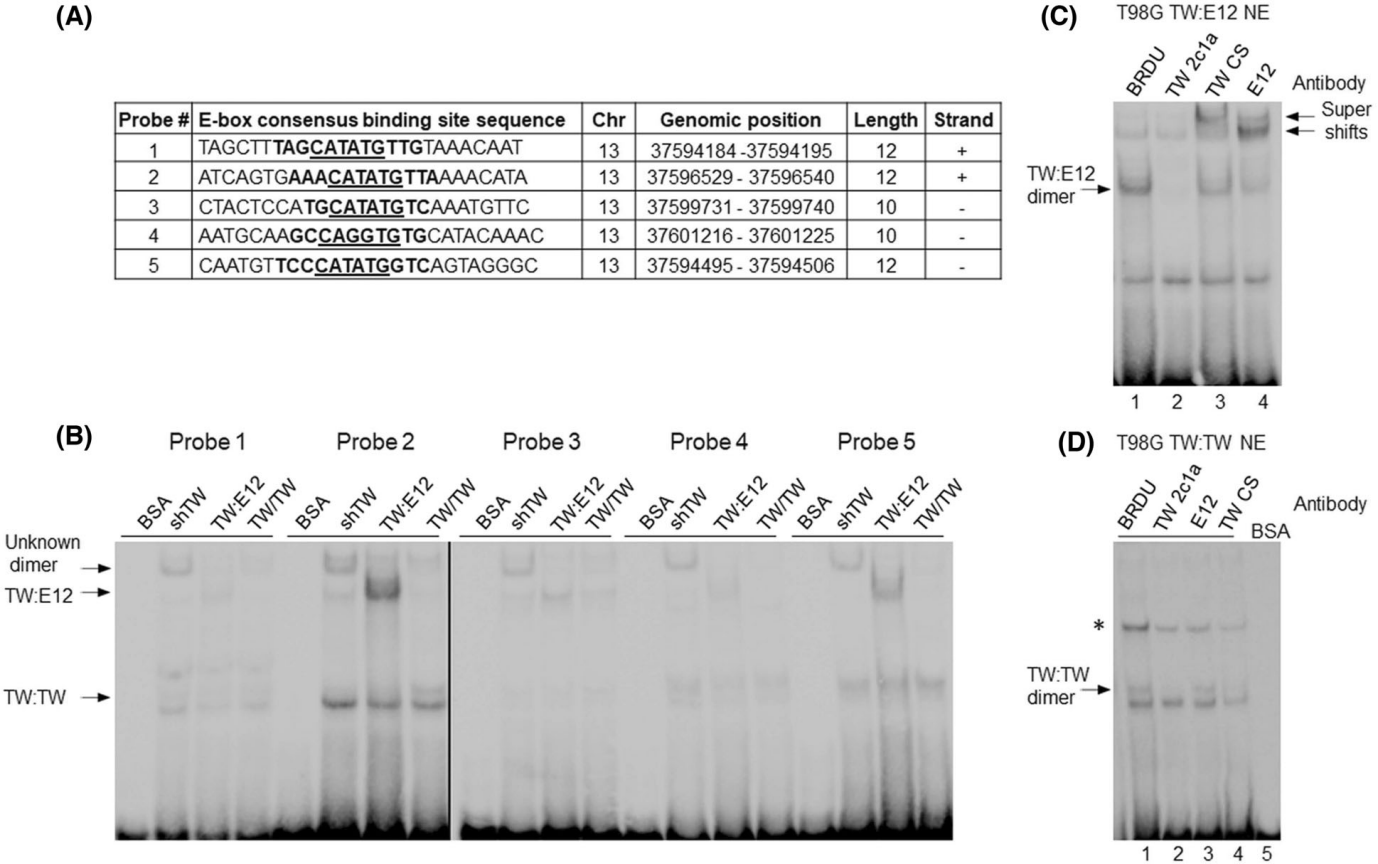
Validation of E‐box sequences identified within periostin (POSTN) for binding to TWIST1 (TW). (A) TW consensus binding sites (E‐Box) located ±5 Kb from transcriptional start site of genomic POSTN sequence with evidence of TF binding derived from publicly available ENCODE DNase I hypersensitivity DNA‐seq data. Identified DNase I footprint, shown in bold, harbored E‐box consensus binding site CANNTG (underlined). An additional 6–9 nucleotides of flanking genomic sequences were added to the footprint on each side. Entire shown sequences were used to synthesize double‐stranded biotinylated probes for electromobility shift assay (EMSA). (B) Nuclear extracts (NE) were derived from T98G cells with TW knockdown (shTW), overexpression of TW:E12 or TW:TW forced dimerization constructs. Bovine serum albumin (BSA) was used as a negative control. Probe 2 demonstrated the strongest signal and, therefore, was used in subsequent experiments. Arrows indicate unique bands that correspond to positions of TW:E12 heterodimer, TW:TW homodimer, and novel protein DNA complex formed under conditions of TW knockdown. Two parallel gels are shown. (C) To demonstrate the identity of proteins bound to the probe, we used two independent TW antibodies and E12 polyclonal antibody added to the binding reaction. TW antibody clone 2C1a (Lane 2 vs. 1) degraded protein DNA complex. Rabbit TW antibody (Cell Signaling Technology, Danvers, MA, USA) and E12 antibody (Santa Cruz; Lanes 3 and 4 vs. 1) formed supershifts and reduced the intensity of the main band confirming TW:E12 dimer binding to the probe. (D) TW antibodies confirmed TW homodimer binding to the DNA probe, resulting in degradation of specific band (Lanes 2 and 4 vs. 1 and 3). No alteration of binding pattern was detected with E12 antibody (Lane 3 vs. 1). Top band (*) was not reproducibly detected. Representative results of three independent experiments are shown in panels (B–D).

To identify candidate TW E‐boxes in the POSTN promoter, we analyzed DNase I footprints in the ENCODE database deduced as evidence of TF occupancy in human brain tissue [20]. Within the ±5 K bp from the POSTN transcriptional start site, we found five unique candidate E‐box consensus recognition sequences harboring common element, CANNTG, that demonstrated TF binding (Fig. 1A). Using electromobility shift assay (EMSA), we tested the TW binding capability of oligonucleotide probes for each identified site (Fig. 1B). In the first experiment, we utilized nuclear extracts from previously described T98G cells expressing TW homodimer or TW:E12 heterodimer FDCs and TW knockdown as a control [2, 18]. The TW:E12 FDC formed a unique band with each probe, albeit with variable intensity, while the TW:TW FDC only formed a unique band with Probe 2 (Fig. 1B). In T98G cells with TW knockdown, each probe uniformly detected a DNA‐protein complex that was negligible or undetectable in nuclear extracts from T98G cells expressing TW:TW or TW:E12 FDCs (Fig. 1B). Overall, Probe 2 provided the most robust DNA‐protein complex formation and was used for all subsequent experiments. Next, using TW and E12 antibodies with EMSA reactions, we confirmed the expected heterodimer and homodimer binding to the DNA probe (Fig. 1C). Mouse monoclonal TW antibody (TW 2C1a) degraded the candidate DNA‐protein complex, while rabbit polyclonal TW antibody (TW CS) and E12 antibody (Santa Cruz, Dallas, TX, USA) resulted in a supershift formation, supporting TW:E12 heterodimer binding (Fig. 1C). Similarly, we confirmed TW homodimer interaction with Probe 2 through differential disruption of probe binding by the TW and E12 antibodies (Fig. 1D). Together these data confirmed TW dimer binding to E‐box sites in the POSTN promoter with preference for site 2. In addition, an unknown protein or protein complex was detected upon TW knockdown at all sites, which, like TW dimers, was most prominently detected by Probe 2.

### 3.2 Novel TCF4 and TCF12 heterodimer binds to E‐box

**Fig. 2:**
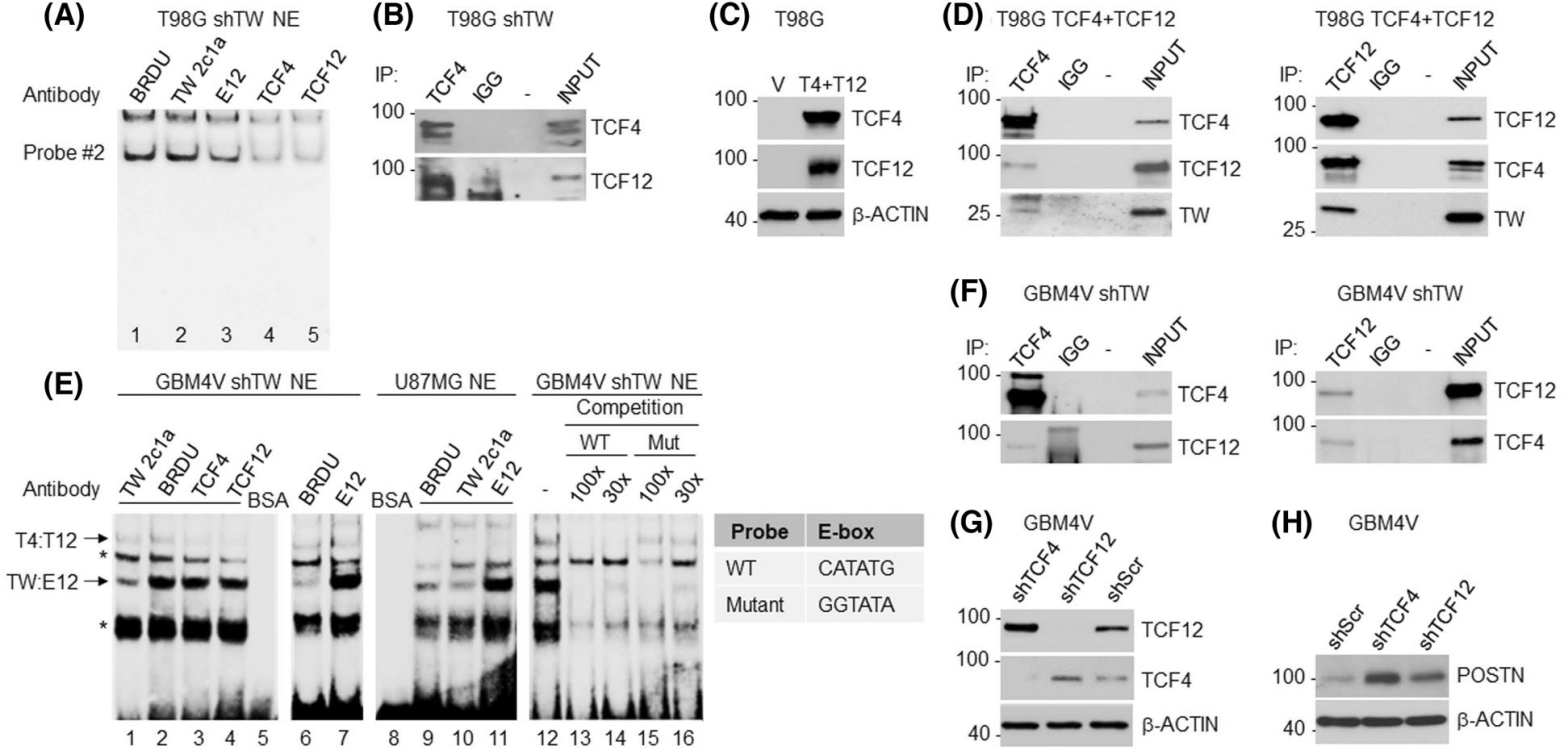
Novel TCF4 and TCF12 heterodimer is detected under conditions of TW knockdown. (A) EMSA analysis of nuclear extracts (NE) from T98G cells with TW knockdown. Antibodies to TCF4 or TCF12 reduce band intensity (Lanes 4 and 5 vs. 1). (B) Co‐immunoprecipitation of endogenous TCF4 protein with TCF12. (C) Confirmation of co‐overexpression of hygromycin‐resistant TCF4(T4) and puromycin‐resistant TCF12 (T12) lentiviral expression constructs in the same cells. Because of high overexpression levels, endogenous signal in vector control (V) is not detected. (D) Protein lysates from C were used to demonstrate co‐IP of TCF4 with TCF12. The lower panel demonstrates co‐IP TCF4 with TW. Co‐IP of TCF12 with TCF4 and TW is depicted on the right panel. (E) EMSA analysis of TW:E12 and TCF4:TCF12 dimer binding in nuclear extracts derived from GBM4V cells with partial TW knockdown (shTW) and parental U87MG cells. TW antibody supplemented binding reaction reduced band intensity compared with BRDU control (Lines 1 vs. 2, and Lines 10 vs. 9). The antibody against E12 increased same band intensity (arrow TW:E12, Lines 7 vs. 6 and Lines 11 vs. 9). TCF4 or TCF12 antibody in the binding reaction reduced the intensity of slower migrating bands (arrow T4:T12, Lines 3 and 4 vs. 2) in GBM4V cells with partial TW knockdown. Together, these results confirmed detection of endogenous TW:E12 and TCF4:TCF12 heterodimers bound to the same sequence (arrows). In the competition experiment (Lines 12–16), a 30‐ or 100‐fold excess of WT unlabeled oligonucleotide efficiently competed for binding to both heterodimers, reducing signal intensity (Lines 13, 14 vs. 12). The mutant oligonucleotide failed to compete for TCF4:TCF12 heterodimer but demonstrated binding to TW:E12 heterodimer (Lines 15, 16 vs. 12). Mutation in WT CANNTG consensus sequence is shown in a table. *—nonspecific/unknown identity band. Independent EMSA panels are aligned to match band pattern. (F) Reciprocal co‐IP of TCF4 and TCF12 proteins from GBM4V cells with partial KD of TW. (G) Confirmation of TCF4 or TCF12 knockdown after infection with corresponding shRNA in GBM4V. (H) Activation of POSTN expression in cells with TCF4 or TCF12 knockdown in GBM4V. Representative results of two independent experiments are shown.

The formation of specific bHLH dimers is regulated in part by the stoichiometry of potential binding partners [26]. Therefore, under conditions of TW knockdown, it was reasoned that altered stoichiometry of TW and its canonical binding partners would promote the formation of alternative bHLH dimers capable of engaging the TW E‐box sites. Because E12, TCF4, and TCF12 are the main bHLHs known to interact with TW [18, 27, 28], we hypothesized that they might form dimers in response to stoichiometric changes after knockdown of TW expression. To test this hypothesis and identify the unknown protein/protein complex bound to the E‐box, we used nuclear extracts from T98G cells with TW knockdown. Binding reactions were supplemented with antibodies to TW, E12, TCF4, or TCF12 while the BRDU antibody served as a negative control (Fig. 2A). Binding of the unknown protein to Probe 2 was decreased by either the TCF4 or TCF12 antibodies, while no effect was detected using the TW or E12 antibodies (Fig. 2A). These results suggested that protein complexes containing TCF4 and TCF12 were binding to the POSTN E‐box site. To determine whether the binding protein might be a TCF4:TCF12 heterodimer, we performed co‐immunoprecipitation and detected endogenous TCF4 interacting with TCF12, supporting the notion that the unknown protein was a TCF4:TCF12 heterodimer (Fig. 2B). To confirm these results, we co‐expressed TCF4 and TCF12 proteins using independent vectors in T98G cells (Fig. 2C) and verified the formation of TCF4:TCF12 heterodimers from the exogenously expressed TCF4 and TCF12 (Fig. 2D). In the same protein preparations, we observed TCF4 and TCF12 binding to endogenous TW (Fig. 2D). Together, these results identified TCF4:TCF12 as a novel heterodimer with shared binding capacity to the same E‐box sites as TW dimers in the POSTN promoter.

Next, we tested whether endogenous TCF4:TCF12 and TW:E12 heterodimers compete for the same E‐box in primary glioma GBM4V with partial TW KD and parental U87MG cells. Using TW and E12 antibodies, we identified bands corresponding to the endogenous TW:E12 heterodimer that is bound to the DNA probe (Fig. 2E, Lines 1–11). While the TW antibody reduced signal intensity due to degradation of protein binding, the E12 mouse monoclonal antibody produced a dramatic increase in heterodimer binding. These results suggested that the E12 antibody used in this study could act as a co‐factor, stimulating TW:E12 heterodimer binding to the DNA. TCF4 and TCF12 antibodies identified heterodimer binding in GBM4V cells with partial TW KD. We were not able to reliably detect TCF4:TCF12 heterodimer in parental U87MG cells although TCF4 and TCF12 proteins were detected on the western blot, albeit in lower levels compared with GBM4V cells (data not shown). These results showed that in primary glioma cells, TW:E12 and TCF4:TCF12 heterodimers compete for the same sequence. To test the requirement of the E‐box consensus binding site for TCF4:TCF12, we performed a competition experiment with excess of WT or mutant unbiotinylated double‐stranded nucleotides corresponding to probe 2 (Fig. 2E, Lines 12–16). The WT probe efficiently competed with TCF4:TCF12 and TW:E12 binding as expected. In contrast, the mutant probe failed to compete with TCF4:TCF12 dimer. However, the TW:E12 dimer tolerated mutations in the E‐box consensus binding site. These results suggested that the CANNTG sequence is required for TCF4:TCF12 dimer binding. Previously, interaction of TW dimers with sequences lacking CANNTG sequence and the role of flanking sequences was demonstrated [29]. To confirm TCF4:TCF12 dimer formation, we performed reciprocal co‐IP using proteins from GBM4V cells with partial TW KD (Fig. 2F).

Because inactivation of TW expression leads to tumor growth inhibition [1] and correlates with increased TCF4:TCF12 heterodimer binding to the E‐box probe (Fig. 2A), we hypothesized that the TCF4:TCF12 heterodimer may function to inhibit POSTN expression by opposing transcriptional activity of the TW:E12 heterodimer. If correct, TCF4 or TCF12 knockdown and reduction of TCF4:TCF12 heterodimer formation would permit E‐box access to TW:E12, thereby activating POSTN expression. To test this hypothesis, we knocked down TCF4 or TCF12 in GBM4V primary GSCs with known TW and POSTN dependent tumorigenicity [16] (Fig. 2G). Consistent with our hypothesis, knockdown of either TCF4 or TCF12 increased POSTN expression (Fig. 2H) compared with the control. Together these experiments suggest that the TCF4:TCF12 heterodimer occupancy of a key TW regulatory element in the POSTN promoter acts as a negative regulator of TW‐dependent POSTN expression.

### 3.3 Overexpression of TCF4 and TCF12 heterodimer inhibits tumor growth and POSTN expression

**Fig. 3:**
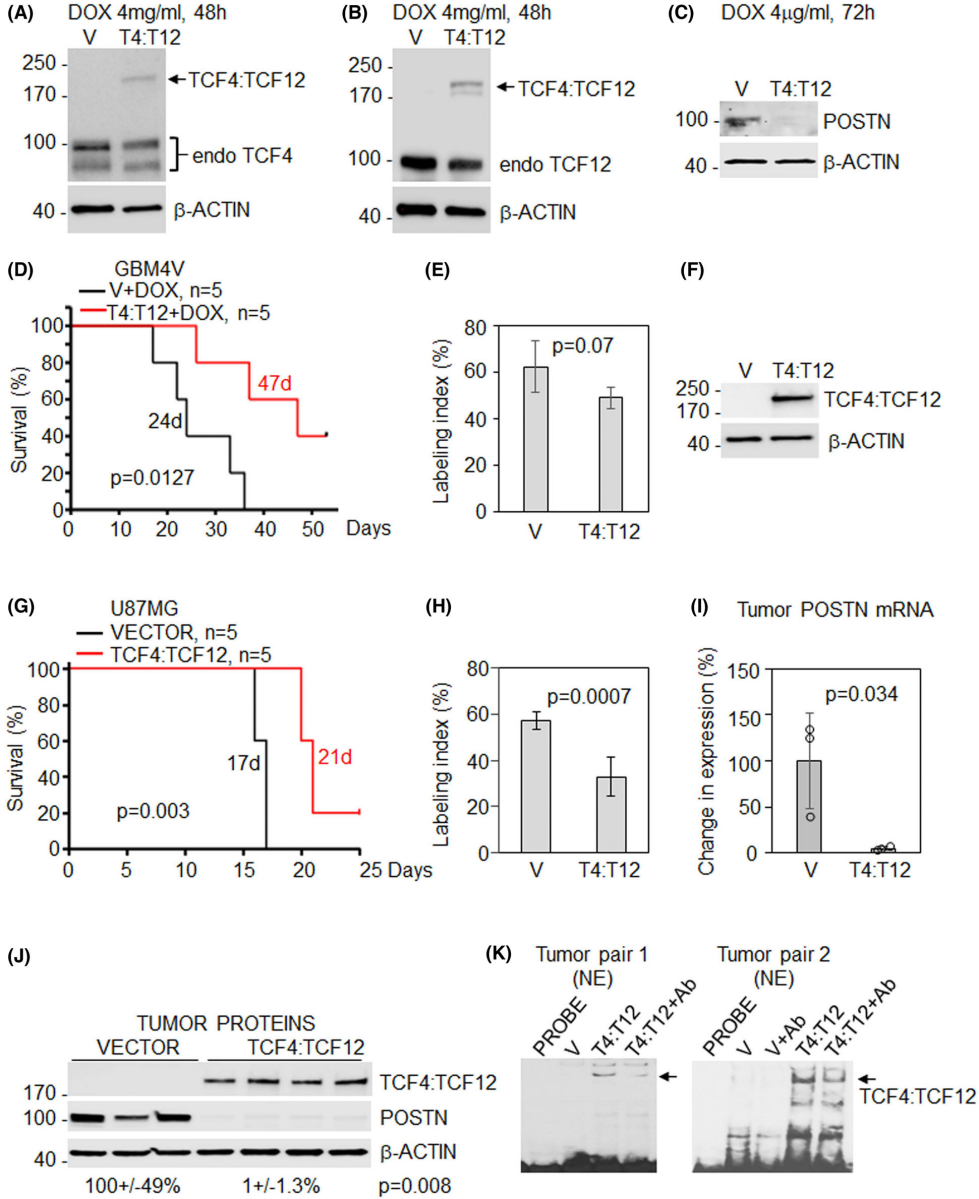
Tumor‐suppressive effect of TCF4:TCF12 forced dimerization construct expression in glioma stem cells GBM4V (A–E) and U87MG (F–K). (A, B) Confirmation of TCF4:TCF12 (T4:T12) forced dimerization construct overexpression induced by Doxycycline (DOX) on the level below endogenous TCF4 and TCF12 in GBM4V cells using corresponding antibodies. (C) Cell treatment with DOX resulted in inhibition of POSTN expression in cells with TCF4:TCF12 FDC overexpression. (D) A significant increase of survival in animals injected with GBM4V cells expressing DOX regulated TCF4:TCF12 FDC compared with vector control (median survival 47 vs. 24 days from starting DOX supplemented food, log‐rank test: *P* = 0.0127). DOX was delivered with food for the duration of experiment. (E) Tendency to reduced Ki67 expression in tumors derived from GBM4V cells with TCF4:TCF12 dimer expression compared with control (V). (F) Confirmation of stable TCF4:TCF12 (T4:T12) dimer expression in U87MG cells using TCF4 and TCF12 (not shown) antibody. (G) A significant survival improvement in animals with implanted U87MG cells with stable TCF4:TCF12 FDC overexpression compared with vector control (median survival 21 vs. 17 days from experiment initiation, log‐rank test: *P* = 0.003). (H) Reduced Ki67 expression in tumors derived from U87MG cells with TCF4:TCF12 dimer overexpression compared with vector (V) control. (I) Analysis of terminal tumors demonstrated inhibition of *POSTN* mRNA expression in tumors with TCF4:TCF12 FDC overexpression (*n* = 3) compared with control (*n* = 3). Results in panels (E, H, and I) are presented as Mean ± SD. Two‐tailed *t*‐test was used to compare the difference between groups. (J) Inhibition of POSTN protein expression in tumors with TCF4:TCF12 FDC overexpression (*n* = 4) compared with vector control (*n* = 3). Protein densitometry in percent is shown as Mean ± SD. Protein expression correlates with levels of mRNA expression shown in panel (I). (K) Confirmation of TCF4:TCF12 dimer binding to the E‐box binding site (Probe 2) in nuclear extracts (NE) from two random pairs of tumors derived from U87MG cells with TCF4:TCF12 (T4:T12) FDC overexpression compared with vector controls. Mix of TCF4 and TCF12 antibodies (Ab) partially degraded DNA‐dimer complex (arrow) in EMSA. BRDU antibody was used as control in other samples. Proteins, mRNA, and nuclear extracts were extracted from randomly selected tumor fragments obtained from tumor bearing animals shown in panel (G). Representative results of three independent experiments are shown in panels (A–C and I and K). Representative results of two independent experiments are shown for panel (F). For panels (E and H), at least 800 cells were counted per representative tumor section in each group (*n* = 3).

We previously showed that loss of POSTN function inhibits GBM tumorigenicity [16] and have provided evidence here that TCF4:TCF12 inhibits POSTN. Therefore, to determine whether TCF4:TCF12 heterodimers would phenocopy the effect of POSTN inhibition in GBM tumors, we generated orthotopic GBM xenograft tumors using human GBM cells overexpressing TCF4:TCF12 FDC. We chose FDC since the overexpression of individual TCF4 or TCF12 proteins could interact with other endogenous bHLHs (e.g., TW and E12) and confound the interpretation of any TCF4:TCF12 heterodimer‐specific effects. In primary human GBM4V cells, we overexpressed a doxycycline (DOX) inducible construct with the TCF4:TCF12 FDC or empty vector using lentiviral transduction. DOX treatment activated TCF4:TCF12 heterodimer expression at levels below endogenous expression of TCF4 and TCF12 (Fig. 3A,B). We reasoned that low levels of transgene activation would mitigate any potential nonphysiological confounding effects from massive overexpression. Furthermore, activation of the TCF4:TCF12 heterodimer was sufficient to inhibit POSTN expression (Fig. 3C). Next, animals were injected with GBM4V cells expressing a DOX‐inducible control vector or TCF4:TCF12 transgene and maintained on a DOX diet for the duration of the experiment. Animals bearing DOX‐inducible TCF4:TCF12 GBM4V cells had markedly improved survival compared with animals bearing DOX‐inducible empty vector (Fig. 3D; *P* = 0.0127). This result is consistent with a potential tumor‐suppressive activity of the TCF4:TCF12 heterodimer in opposition to the oncogenic effects of POSTN. We found that tumors with TCF4:TCF12 dimer activation showed tendency to reduced cell proliferation measured by Ki67 labeling index (Fig. 3E).

To confirm these findings in a second GBM model, we stably overexpressed TCF4:TCF12 FDC under control of a stronger cytomegalovirus promoter in U87MG cells (Fig. 3F). The rationale for utilizing U87MG is their low endogenous expression of TCF4 and TCF12 protein compared with GBM4V cells (not shown) and TW‐dependent regulation of POSTN expression *in vivo* only [1]. Therefore, U87MG enables us to evaluate TCF4:TCF12 dimer expression effects while minimizing any confounding impact from endogenous proteins. Animal survival was significantly extended in animals injected with U87MG cells expressing the TCF4:TCF12 dimer versus vector control animals (median 21 vs. 17 days; *P* = 0.003; Fig. 3G). Delay of tumor growth correlated with significantly reduced Ki67 labeling index in tumors derived from cells with TCF4:TCF12 expression compared with control cells (Fig. 3H and Fig. S1). Our analysis of terminal tumors showed that TCF4:TCF12 dimer expression efficiently inhibited *POSTN* mRNA and protein expression (Fig. 3I,J) and activated binding of the TCF4:TCF12 dimer to the DNA Probe 2 compared with control tumors (Fig. 3K). Antibody added to the binding reaction confirmed TCF4:TCF12 dimer identity. These results provide important and consistent *in vivo* physiologic evidence for a novel tumor‐suppressive function of the TCF4:TCF12 heterodimer through E‐box binding and repression of POSTN. Thus, TCF4:TCF12 heterodimer activity is similar in primary GBM cells and traditional GBM cell line.

## 4 Discussion

Basic helix–loop–helix transcription factors (bHLH‐TFs) play important roles in regulating many normal and pathologic biological states, including cancer [30]. For instance, we previously demonstrated that the TW is correlated with clinical malignancy and promotes invasion, stemness, and tumorigenicity of GBM cells [1, 2, 24]. TWIST1 form hetero‐ or homodimers that regulate target gene expression by binding to CANNTG E‐box sequences [17, 26, 31]. For example, TW:TW homodimers and TW:E12 heterodimers differentially regulate malignancy [32] and activate target genes like POSTN [18], [27, 33]. The formation and prevalence of different bHLH dimers is a function of multiple factors, including protein–protein and protein–DNA binding affinities [34], post‐translational modifications, and relative abundance or stoichiometry [26, 35]. With over 100 members in the bHLH‐TF family and the frequent occurrence of E‐box consensus binding sites in the mammalian genome [34], the mechanisms regulating the bHLH dimer network are complex and not fully understood. Here, we sought to better understand how specific bHLH‐TF dimer motifs (i.e., TW:E12 and TW:TW) and E‐box sites interact to regulate expression of the TW transcriptional target gene POSTN and identified a novel tumor‐suppressive bHLH‐TF heterodimer, TCF4:TCF12, which functioned in part by competitively inhibiting TW dimer occupancy of E‐boxes and suppressing POSTN expression.

In contrast to TW dimers, the TCF4:TCF12 bHLH heterodimer has not been previously reported. Limited information exists about mechanisms of TCF4 and TCF12 activity and dimerization in cancers, including glioma. Somatic TCF12 mutations are reported in 7.5% of anaplastic oligodendroglioma patients and said mutations inhibit *TCF12* transcriptional activity and increase tumor malignancy [36]. TCF12 alterations were found in GBM (27%) and low‐grade gliomas (18%) and, while not prognostic for survival, low TCF12 expression seems to correlate with unfavorable patient outcomes [37]. In medulloblastomas, somatic TCF4 mutations are detected in 20% of Sonic hedgehog subtype cases [38]. Consistent with its tumor‐suppressive function, low TCF4 expression correlates with worse clinical outcomes while overexpression of TCF4 activates tumor suppressors and postnatal inhibition of TCF4 increases cell proliferation [38]. TCF4 interacts with the bHLH‐TF Olig2 to promote terminal differentiation in the oligodendrocyte lineage [39]. Olig2 is frequently expressed in gliomas [40] and is an important driver of GBM tumorigenicity, genotoxic resistance, and tumor growth *in vivo* (reviewed in [41]). Olig2 inhibition reduces tumor growth *in vivo*. Therefore, it will be of interest to determine whether Olig2 inhibition is associated with TCF4:TCF12 dimer activation that subsequently leads to observed tumor suppression.

The present study identified the competitive engagement of common E‐box sites by tumor‐suppressive TCF4:TCF12 and oncogenic TW:E12 heterodimers as a novel mechanism in the regulation of POSTN expression. Repression of POSTN by TCF4:TCF12 FDC expression inhibited tumorigenicity and extended animal survival. In our model, the TCF4:TCF12 heterodimer, once formed, binds to the E‐box, displaces the TW:E12 dimer, and represses POSTN mRNA expression. The mechanisms of TCF4:TCF12 dimer activation are not known. We found that inhibition of TW expression activated novel TCF4:TCF12 heterodimer binding. Further investigation of the impact of increased protein expression and/or dimer post‐translational modifications (e.g., phosphorylation, which known to regulate TW dimer motifs [18]) may inform novel therapeutic approaches to inhibit TW‐dependent phenotypes. Since TCF4 and TCF12 form heterodimers with TW, we speculate that targeting TW heterodimers will alter bHLH stoichiometry, increase unbounded levels of TCF4 and TCF12, and favor the formation of tumor‐suppressive TCF4:TCF12 heterodimers. Future studies will validate the scope of these mechanisms in additional glioma cell lines, genome‐wide TCF4:TCF12 transcriptional dimer activity. Elucidation of new mechanisms of bHLH interactions, as described here, is critical for better mechanistic understanding of how to leverage the bHLH transcriptional network to inhibit malignancy in GBM and other cancers. These approaches could include stoichiometric modulation of dimer motifs by directly targeting gene expression, steric disruption of protein–protein or protein–DNA binding by small molecules, or indirectly through modulation of post‐translation modifications that impact dimerization.

## 5 Conclusion

The oncogenic functions of TW, a bHLH transcription factor and master regulator of mesenchymal phenotypes, are mediated through differential functional activity of its dimerization partners. Inhibition of TW expression reduces invasion, stem cell maintenance, metastasis, and tumorigenicity in many cancers. The relationship between inhibition of TW expression and malignancy is thought to derive from reduced formation of pro‐tumorigenic dimers, particularly the TW:E12 heterodimer. This study revisited current dogma and found that inhibition of TW expression also results in activation of a novel dimer composed of two bHLH proteins: TCF4 and TCF12. The TCF4:TCF12 heterodimer occupied the same E‐box binding site in the TW target gene, POSTN, as the pro‐invasive TW:E12 heterodimer. Expression of a tethered TCF4:TCF12 dimer in GBM cells markedly inhibited POSTN expression *in vitro* and extended animal survival *in vivo*. These data together identify a new bHLH heterodimer with novel tumor‐suppressive functions that acts through competitive E‐box binding and displacement of oncogenic TW heterodimers. This new mechanistic insight into regulation of TW‐mediated malignancy has implications for targeting TW function in GBM and many other cancers regulated by TW. Our results validated ENCODE data analysis for targeted identification of transcription factors.

## Conflict of interest

The authors declare no conflict of interest.

## Author contributions

AMM and SAM served as main contributors, collected and assembled the data, and drafted manuscript. CCF performed bioinformatics analysis of ENCODE data. PJH, RCR, and AMM designed and directed this study and provided conceptual guidance, data interpretation, and manuscript writing.

## Supporting information


**Fig. S1.** Representative photomicrograph of Ki67 immunohistochemistry in xenograft tumors derived from U87MG cells expressing empty vector or TCF4:TCF12 dimer.
**Table S1.** List of antibodies used in the study.
**Table S2.** List of DNA constructs used in the study.

## Data Availability

The data that support the findings of this study are available from the corresponding author upon reasonable request.
