# Supplementary material for: Novel TCF4:TCF12 heterodimer inhibits glioblastoma growth
Source: Mol Oncol. 2023 Aug 7;18(3):517–27. doi: 10.1002/1878-0261.13496 (PMC10920085; doi:10.1002/1878-0261.13496)
Supplement: Supplementary file 1 — Fig. S1. Representative photomicrograph of Ki67 immunohistochemistry in xenograft tumors derived from U87MG cells expressing empty vector or TCF4:TCF12 dimer. Table S1. List of antibodies used in the study. Table S2. List of DNA constructs used in the study. [file MOL2-18-517-s001.docx]

**Supplemental file.**

**Novel TCF4:TCF12 heterodimer inhibits glioblastoma growth**

**Svetlana A. Mikheeva, Cory C. Funk, Philip J. Horner, Robert C. Rostomily,**

**Andrei M. Mikheev**

**Fig. S1: Representative photomicrograph of Ki67 immunohistochemistry in xenograft tumors derived from U87MG cells expressing empty vector or TCF4:TCF12 dimer.** Tumor sections were deparaffinized and subjected to antigen retrieval using 10mM sodium citrate, 0.05% Tween 20, pH6.0. After quenching endogenous peroxidase and blocking sections were incubated with Ki67 antibody overnight followed by application of HRP Horse Anti-Rabbit IgG PLUS Polymer (Vector Laboratories, Newark, CA, USA). Sections were counterstained with hematoxylin. Scale bar = 80μM.

| **Table S1. List of antibodies used in the study.** | | | |
| --- | --- | --- | --- |
| Antibody | Catalog # | Source | Manufacturer |
| TWIST1 | sc-81417 | mouse | Santa Cruz Biotechnology, Dallas,TX |
| TWIST1 | 46702 | rabbit | Cell Signaling, Danvers, MA |
| E12 (E2A) | sc-416 | mouse | Santa Cruz Biotechnology, Dallas,TX |
| E12 (E2A) | sc-349 | rabbit | Santa Cruz Biotechnology, Dallas,TX |
| TCF4 (ITF-2) | sc-393407 | mouse | Santa Cruz Biotechnology, Dallas,TX |
| TCF4 (ITF-2) | ab217668 | rabbit | Abcam, Waltham, MA |
| TCF12 (HEB) | sc-28364 | mouse | Santa Cruz Biotechnology, Dallas,TX |
| TCF12 (HEB) | 14419-1-AP | rabbit | Proteintech, Rosemont, IL |
| BrdU | sc-32323 | mouse | Santa Cruz Biotechnology, Dallas,TX |
| β-Actin | A5441 | mouse | Sigma-Aldrich, St. Louis, MO |
| Periostin (POSTN) | AG-20B-0033-C100 | mouse | AdipoGen, San Diego, CA |
| Ki67 | Ab15580 | rabbit | Abcam, Waltham, MA |

| **Table S2. List of DNA constructs used in the study.** | | | | | |
| --- | --- | --- | --- | --- | --- |
| Gene name | Refseq | Clone ID | Resistance | DOX/Stable/knockdown | Manufacturer |
| TCF12 | NM_003205 | TRCN0000274222 | puro | knockdown | Sigma-Aldrich, St. Louis, MO |
| TCF4 | NM_003199 | TRCN0000274214 | puro | knockdown | Sigma-Aldrich, St. Louis, MO |
| shScr | non-silencing shRNA | 1864 | puro | knockdown | Sigma-Aldrich, St. Louis, MO |
| TCF4 | NM_001243226 | N/A | hygro | stable | VectorBuilder, Chicago, IL |
| TCF12 | NM_207037 | N/A | puro | stable | VectorBuilder, Chicago, IL |
| TCF4:TCF12 (FDC) | (NM_001243226):(NM_207037) | N/A | hygro | stable | VectorBuilder, Chicago, IL |
| Control vector (stuffer 300) | N/A | N/A | hygro | stable | VectorBuilder, Chicago, IL |
| Control vector (stuffer 300) | N/A | N/A | puro | stable | VectorBuilder, Chicago, IL |
| Tet-On TCF4:TCF12(FDC) | (NM_001243226):(NM_207037) | N/A | puro | DOX | VectorBuilder, Chicago, IL |
| Tet-On Control (stuffer 300) | N/A | N/A | puro | DOX | VectorBuilder, Chicago, IL |
| rtTA-3G | 3^rd^ generation transactivator | N/A | hygro | stable | VectorBuilder, Chicago, IL |
